# Patterns of Microbiome Variation Among Infrapopulations of Permanent Bloodsucking Parasites

**DOI:** 10.3389/fmicb.2021.642543

**Published:** 2021-04-16

**Authors:** Jorge Doña, Stephany Virrueta Herrera, Tommi Nyman, Mervi Kunnasranta, Kevin P. Johnson

**Affiliations:** ^1^Illinois Natural History Survey, Prairie Research Institute, University of Illinois at Urbana-Champaign, Champaign, IL, United States; ^2^Departamento de Biología Animal, Universidad de Granada, Granada, Spain; ^3^Department of Ecosystems in the Barents Region, Norwegian Institute of Bioeconomy Research, Svanvik, Norway; ^4^Department of Environmental and Biological Sciences, University of Eastern Finland, Joensuu, Finland; ^5^Natural Resources Institute Finland, Joensuu, Finland

**Keywords:** genome-resolved metagenomics, host-symbiont, intraspecific variation, lice, microbiota, shotgun metagenomics, symbiont

## Abstract

While interspecific variation in microbiome composition can often be readily explained by factors such as host species identity, there is still limited knowledge of how microbiomes vary at scales lower than the species level (e.g., between individuals or populations). Here, we evaluated variation in microbiome composition of individual parasites among infrapopulations (i.e., populations of parasites of the same species living on a single host individual). To address this question, we used genome-resolved and shotgun metagenomic data of 17 infrapopulations (balanced design) of the permanent, bloodsucking seal louse *Echinophthirius horridus* sampled from individual Saimaa ringed seals *Pusa hispida saimensis*. Both genome-resolved and read-based metagenomic classification approaches consistently show that parasite infrapopulation identity is a significant factor that explains both qualitative and quantitative patterns of microbiome variation at the intraspecific level. This study contributes to the general understanding of the factors driving patterns of intraspecific variation in microbiome composition, especially of bloodsucking parasites, and has implications for understanding how well-known processes occurring at higher taxonomic levels, such as phylosymbiosis, might arise in these systems.

## Introduction

Patterns of inter- and intraspecific variation in microbiome composition of animals have received much attention because the microbiome may influence many biological processes that have considerable effects on the host (Clemente et al., 2012; Le Chatelier et al., 2013; Rothschild et al., 2018; Rudman et al., 2019; Velazquez et al., 2019). For instance, particular microbiome compositions have been found to drive genomic adaptation (Rudman et al., 2019) or to confer protection against pathogens (Velazquez et al., 2019).

In general, both stochastic (e.g., dispersal, or ecological drift) and deterministic (e.g., host immunological regulation, or microbe–microbe interactions) processes operate across multiple spatial scales to shape the composition of animal microbiomes (Adair and Douglas, 2017; Kohl, 2020). In particular, among the many determinants shaping microbiome composition, host species identity has been repeatedly identified as a key factor determining the composition of animal microbiomes (Brooks et al., 2016; Mazel et al., 2018; Nishida and Ochman, 2018; Knowles et al., 2019; Lutz et al., 2019; Lim and Bordenstein, 2020; Song et al., 2020). In other words, microbiomes of individuals of the same species tend to be more similar than to those of another species. This pattern is generally the result of filtering microbial taxa by the host (e.g., through host diet, habitat, or immune system, Adair and Douglas, 2017) or result from host–microbe coevolution (Lim and Bordenstein, 2020). When this process exhibits phylogenetic signal, the pattern is known as phylosymbiosis (i.e., microbial community relationships that recapitulate the phylogeny of their host, Brucker and Bordenstein, 2013; Brooks et al., 2016; Lim and Bordenstein, 2020). Nonetheless, several aspects of the variation of animal microbiomes are yet to be better understood (Lim and Bordenstein, 2020). In particular, for non-human animals, there is still much to learn about how microbiomes vary at scales below the species level, such as between populations (Blekhman et al., 2015; Kohl et al., 2018; Rothschild et al., 2018; Campbell et al., 2020; Fountain-Jones et al., 2020) or ecotypes (Agany et al., 2020).

An area of focus on understanding intraspecific variation in microbiome composition has been bloodsucking parasites. In these parasites, previous studies have consistently found a major role of the host species in shaping microbiome composition in the parasites (Osei-Poku et al., 2012; Zhang et al., 2014; Swei and Kwan, 2017; Zolnik et al., 2018; Díaz-Sánchez et al., 2019; Landesman et al., 2019; Lee et al., 2019; Muturi et al., 2019). However, in ticks (*Ixodes scapularis*), host individual identity of the blood meal was even more important than host species identity in explaining microbiome composition (Landesman et al., 2019). These results suggested that individual host identity of the blood meal might be an important factor that shapes parasite microbiomes at the intraspecific level (Landesman et al., 2019). In theory, microbiomes of individual bloodsucking parasites could vary due to: (1) the individual parasite immune system that may impose selection on different bacterial taxa (Blekhman et al., 2015; Suzuki et al., 2019); (2) differences in the source of the blood meal that may transfer or disperse particular bacterial taxa, or modulate bacteria by creating specific conditions during digestion (Rothschild et al., 2018); (3) microbe–microbe interactions (Hassani et al., 2018); and (4) stochastic processes (e.g., ecological drift) (Lankau et al., 2012). However, for most species, and for bloodsucking parasites in particular, the nature of intraspecific variation in microbiomes and the relative importance of factors shaping this variation remain understudied.

Sucking lice (Phthiraptera: Anoplura) are permanent blood-feeding ectoparasites that live in the fur or hairs of mammals. Anopluran lice have been shown to host intracellular bacterial endosymbionts that are likely to help to complement deficiencies in their diet, and these symbionts tend to be located on specialized structures known as mycetomes (Buchner, 1965; Boyd and Reed, 2012; Sasaki-Fukatsu et al., 2006; Perotti et al., 2007, 2008). Previous studies have found that members of Anoplura host a single endosymbiont, but belonging to different bacterial genera depending on louse species, including *Riesia* (Sasaki-Fukatsu et al., 2006; Kirkness et al., 2010; Boyd et al., 2014), *Sodalis* (Boyd et al., 2016), and *Legionella* (Říhová et al., 2017). We also know from these studies that, as in other arthropod endosymbionts, louse endosymbionts tend to have reduced genomes (Kirkness et al., 2010; Boyd et al., 2017). On the other hand, processes such as replacement (i.e., the substitution of one endosymbiotic species by another) and independent acquisitions of different endosymbionts can occur across evolutionary time scales (i.e., millions of years) (Sasaki-Fukatsu et al., 2006; Allen et al., 2007, 2016; Hypša and Køížek, 2007; Fukatsu et al., 2009). Thus, while there is some background knowledge on louse endosymbionts, several aspects are yet to be understood. For instance, how microbiomes vary across organs, systems, or individuals of lice from the same species is mostly unknown, with a single study to date providing bacterial community data for different individual lice of the same species (Říhová et al., 2019).

The sucking lice of pinnipeds (seals, sea lions, and walrus) are of particular interest because of their need to adapt to the aquatic lifestyle of their hosts (Durden and Musser, 1994; Leonardi et al., 2013). There is evidence that the sucking lice of seals and sea lions have codiversified with their hosts (Kim, 1971, 1975, 1985; Leonardi et al., 2019). Indeed, the sucking lice of pinnipeds represent an interesting system in which to study the variation in microbiome composition and the drivers of this variation at an intraspecific level because: (1) these lice have well defined, isolated populations (infrapopulations) on individual seal hosts, due to an expected low rate of horizontal dispersal among host individuals, which is only possible during the seals’ haul-out periods on land or ice (Kim, 1985; Leonardi et al., 2013, 2019); and (2) these lice feed only upon the blood of their host (Snodgrass, 1944; Kim, 1985), so that it can be assumed that individuals from the same infrapopulation feed upon “exactly” the same resource (i.e., the blood of the individual seal on which they occur). In addition, previous studies conducted on seal microbiomes have found that while factors such as species identity, age, sex, and diet play a role in shaping seal microbial communities, seals show evidence of a core microbiome with which they have co-evolved (Nelson et al., 2013; Acquarone et al., 2020; Kim et al., 2020; Stoffel et al., 2020).

Here, we used genome-resolved approaches (the construction of draft microbial genomes from short-read shotgun sequencing data; Bowers et al., 2017; Uritskiy et al., 2018) and metagenomic classification tools (taxonomic classification of individual sequencing reads; Menzel et al., 2016) to infer patterns of microbiome variation among individuals of the sucking seal louse *Echinophthirius horridus* (von Olfers, 1816) inhabiting individual Saimaa ringed seals *Pusa hispida saimensis* (Nordquist, 1899). These two approaches have different limitations and strengths. For example, the genome-resolved approach allows the assembly of multiple highly complete bacterial genomes, but only for organisms with enough coverage to be assembled and binned. On the other hand, metagenomic classification of reads may offer a more comprehensive picture of community composition because of higher database completeness or less strict thresholds to analyze data. However, read classification is limited by the fact that it is based only on the fraction of reads that map to reference databases (Quince et al., 2017). Our sampling design, involving analysis of two individual lice from each of 17 seals, allowed us to evaluate the degree to which variation in microbiome composition among individual lice is explained by the infrapopulation (the identity of the seal host).

## Materials and Methods

### Sampling, DNA Extraction, and Sequencing

Thirty-four individual lice were sampled from 17 individual Saimaa ringed seals (*Pusa hispida saimensis*), which is an endemic endangered landlocked subspecies of the ringed seal living in freshwater Lake Saimaa in Finland (e.g., Nyman et al., 2014). Individual lice were collected from seals found dead or from seals that were live-captured for telemetry studies (e.g., Niemi et al., 2019), and placed in 2-ml screw-cap tubes with 99.5% ethanol. Lice from a single seal individual were put in the same tube. Prior to DNA extraction, each louse individual was rinsed with 95% ethanol and placed alone in a new sterile vial; then, the remaining ethanol was evaporated at room temperature.

Whole lice were ground up individually, and genomic DNA was extracted using the Qiagen QIAamp DNA Micro Kit (Qiagen, Valencia, CA, United States). The standard protocol was modified so that specimens were incubated in ATL buffer and proteinase K at 55 (insert degree) C for 48 h instead of the recommended 1–3 h, as well as by substituting buffer AE with buffer EB (elution buffer). This was done to ensure maximal yield (greater than 5 ng) of DNA from each louse. Each DNA extract was quantified with a Qubit 2.0 Fluorometer (Invitrogen, Carlsbad, CA, United States) following the manufacturer’s recommended protocols.

Shotgun genomic libraries were prepared from the extracts with Hyper Library construction kits (Kapa Biosystems, Wilmington, MA, United States). The libraries were quantitated by quantitative PCR (qPCR) on a Bio-Rad CFX Connect Real-Time System (Bio-Rad Laboratories, Inc., CA, United States) and 150 bp pair-end sequenced on either one of two lanes (Supplementary Table 1) of an Illumina NovaSeq 6000 sequencer (Albany, New York, United States). FASTQ files from sequence data were generated and demultiplexed with bcl2fastq v.2.20. All library preparations, sequencing, and FASTQ file generation were carried out at the Roy J. Carver Biotechnology Center (University of Illinois, Urbana, IL, United States). Raw reads were subsequently deposited to the NCBI GenBank SRA database (Supplementary Table 1).

### Metagenomic Analyses

For the genome-resolved metagenomic analyses, we used the metaWRAP v1.1.5 pipeline (Uritskiy et al., 2018) along with all the recommended databases (i.e., Checkm_DB, NCBI_nt, and NCBI_tax). We used the metaWRAP Read_qc module with default parameters to quality trim the reads and to de-contaminate each sample from host reads. For decontamination, we ran a *de novo* genome assembly of an individual louse of the same species, not included in this study, and with a high amount of sequencing data (“Echor52”) in Abyss v2.0.1 (Jackman et al., 2017). Finally, we filtered out all non-bacterial reads from the contig file using Blobtools v1.0.1 (Laetsch and Blaxter, 2017) and used this file to decontaminate all the other samples with the metaWRAP Read_qc module. See Supplementary Table 1 and Data Availability section for more details on the data preprocessing results. Next, we co-assembled reads from all the samples with the metaWRAP Assembly module (–use metaspades option) (Nurk et al., 2017). For this assembly, and because of memory limitations, we used BBNorm^1^ before assembly to reduce the coverage of the concatenated FASTQ file to a maximum of 100× and to discard reads with coverage under 3×. We binned reads with the metaWRAP Binning module (–maxbin2 –concoct –metabat2 options) (Alneberg et al., 2014; Wu et al., 2016; Kang et al., 2019) and then consolidated the resulting bins into a final bin set with both metaWRAP’s Bin_refinement module (-c 50 -× 10 options) and the Reassemble_bins module. We quantified the bins resulting from the Bin_refinement module with Salmon (Patro et al., 2017) using the Quant_bins module with default parameters. Finally, we classified bins using the Classify_bins module. This module uses Taxator-tk, which gives highly accurate but conservative classifications (Dröge et al., 2015). Accordingly, we also uploaded our final metagenome-assembled genomes (MAGs) to MiGA for a complementary analysis to determine the most likely taxonomic classification and novelty rank of the bin (Rodriguez-R et al., 2018). We used the NCBI Genome database (Prokaryotes; February 26, 2020 version) for this analysis.

For the metagenomic classification of reads, we used the metagenomic classifier Kaiju (Menzel et al., 2016) with Reference database: nr (Bacteria and Archaea; Database date: 2017-05-16). We used the default parameters for these analyses (SEG low complexity filter: yes; Run mode: greedy; Minimum match length: 11; Minimum match score: 75; Allowed mismatches: 5). We then converted Kaiju’s output files into a summary table at the genus and species level and filtered out taxa with low abundances (<0.1% of the total reads). We also removed poorly identified taxa because they would artificially increase the similarity between our samples. Specifically, the following taxa were excluded: “NA,” “Viruses,” “archaeon,” “uncultured bacterium,” “uncultured Gammaproteobacteria bacterium” (Supplementary Tables 2, 3).

Lastly, we used Decontam v1.2.1 to filter out bacterial taxa exhibiting known statistical properties of contaminants (Davis et al., 2018). We used the frequency method (*isContaminant* function) which is based on the inverse relationship between the relative abundance of contaminants and sample DNA concentration, and also has been found suitable for samples dominated by host DNA (Willner et al., 2012; Lusk, 2014; Salter et al., 2014; Jervis-Bardy et al., 2015; Hooper et al., 2019; McArdle and Kaforou, 2020). As input for Decontam analyses, we used the aforementioned total DNA concentration values. Then, as recommended, we explored the distribution of scores assigned by Decontam to assign the threshold according to bimodality between very low and high scores (Davis et al., 2018). For the MAGs matrix, no bimodality was found, and thus we used the 0.1 default value (Supplementary Figure 1A). None of the MAGs were classified as contaminants, according to Decontam. For Kaiju matrices, a 0.3 threshold value was selected for the species-level matrix (Supplementary Figure 1B) and 0.31 for the genus-level matrix (Supplementary Figure 1C). Decontam filtered out a single species (*Clostridia* bacterium k32) from the species matrix and two genera (*Cupriavidus* and *Massilia*) from the genus matrix.

### Statistical Analyses

For the genome-resolved metagenomic analyses, we used the normalized MAGs compositional matrices resulting from Salmon. Specifically, these MAG counts are standardized to the individual sample size (MAG copies per million reads) and thus allow between-sample comparisons. For the Kaiju analyses, we used the rarefy_even_depth function of phyloseq (without replacement as in the hypergeometric model) to rarefy samples to the smallest number of classified sequences per individual observed (85,513 and 71,267 reads in genus and species matrices, respectively) (Weiss et al., 2017).

To visualize similarities of microbiome composition among louse individuals from the same or different individual seal hosts, we constructed non-metric multidimensional scaling (NMDS) ordinations based on Bray–Curtis and Jaccard (binary = T) dissimilarities using the phyloseq v1.26-1 R package (McMurdie and Holmes, 2013). Also, to remove subjective bias when interpreting the results of our main NMDS ordinations, we also analyzed whether Bray–Curtis dissimilarity distances between samples from the same infrapopulation were lower than those of comparisons with samples from other infrapopulations. We statistically tested these results using two-sided Sign tests, with which we evaluated whether the difference between within- versus among-infrapopulation medians was significantly different from 0.

To assess the influence of individual host identity on the microbiome composition of louse individuals, we conducted a permutational multivariate analysis of variance (PERMANOVA) (Anderson and Walsh, 2013; Anderson, 2014). PERMANOVA analyses were done using the *adonis2* function in vegan v2.5–4 (Oksanen et al., 2019), based on Bray–Curtis and Jaccard distance matrices with 100 iterations. In PERMANOVA analyses, for the individual host identity factor, our within-group sample size (*n* = 2) was smaller than both the total number of groups (*n* = 17) and the total sample size (*n* = 34). From the perspective of statistical power for testing effects related to host individuals, the relatively high number of hosts, in essence, offsets the low number of lice per host. Nevertheless, to account for a potential deviation in F-statistics and *R*^2^ values (Kelly et al., 2015), we wrote an R simulation that randomly subsampled the infrapopulations from which the louse came (5 infrapopulations per iteration). We ran 10 iterations and ran a PERMANOVA analysis for each iteration. Note that, for a few iterations, subsampled samples were too similar and PERMANOVA could not be done. In addition, we ran PERMANOVA analyses to explore additional factors (louse sex: male, female; sequencing lane: 1, 2; and host status: dead, alive) that may explain variance in microbiome composition. Furthermore, we included significant factors as the first factors of the host identity PERMANOVA models (i.e., to obtain the variance explained by host identity after accounting for the variance explained by that factor). We also restricted the groups in which permutations could be done to only those with the same value of that vector using the strata argument (e.g., for a sample collected from a dead host, and for the host-status factor, permutations could only be done among other dead hosts). Lastly, we ran a Mantel test using the *mantel* function in vegan (method = spearman, permutations = 9999) to explore if host locality (i.e., the coordinates in which each host was sampled) correlated with the microbiome composition of louse individuals. For this analysis, we ran 10 iterations of an R simulation in which we randomly subsampled one louse sample for each individual host and ran a Mantel test for each iteration. The following packages were used to produce the plots: ggplot2 v3.1.0.9 (Wickham, 2016), grid v3.5.3 (R Core Team, 2019), gridExtra v.2.3 (Auguie, 2016), ggrepel v0.8.0 (Slowikowski et al., 2019), ggpubr v.0.2.5 (Kassambara, 2018), and ggsci v2.9 (Xiao, 2018).

## Results

**FIGURE 1:**
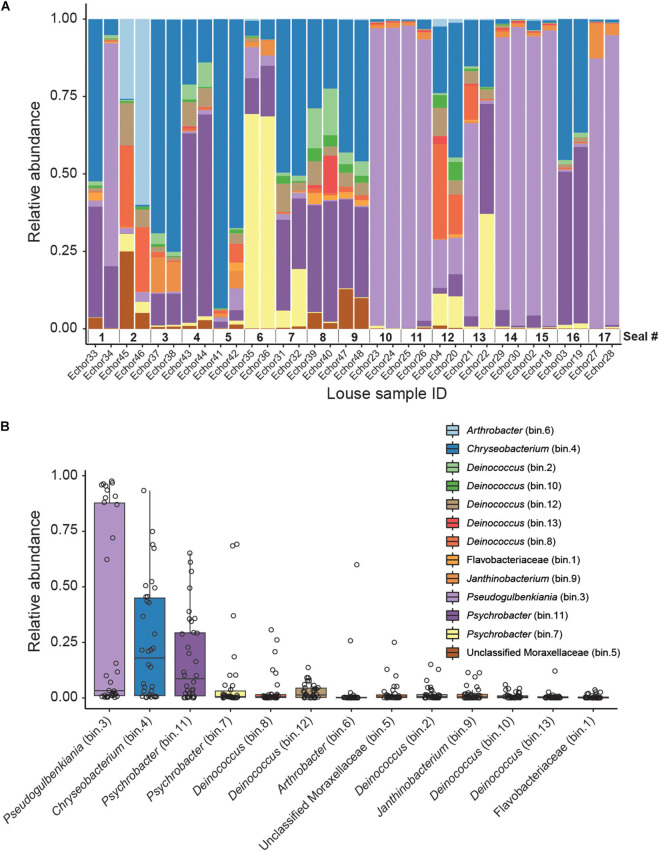
Genome-resolved metagenomic data. **(A)** Stacked bar plot showing the relative abundances of MAGs in each louse sample. Note that samples are ordered according to host (i.e., samples from the same host are next to each other). **(B)** Boxplot summarizing the relative abundance of each MAG across the louse samples. Individual points (horizontally jittered) depict the relative abundance of each MAG in each sample.

From the genome-resolved metagenomics pipeline, 13 high-quality bacterial metagenome-assembled genomes (MAGs) were obtained (Table 1 and Figure 1). According to MiGA analyses, 10 of them (77%) likely belong to a species not represented in the NCBI Genome database.

**TABLE 1:** Statistics of the MAGs assembled.

MAG name	Completeness (%)	Contamination (%)	N50 (bp)	Size (bp)	Taxator tk ID	MiGA ID	RDP ID	Taxonomic novelty
bin.1	100	1.07	57370	1869975	Flavobacteriaceae	Flavobacteriaceae*	NA	Species****
bin.4	99.26	0.24	81315	2500734	Flavobacteriaceae	Chryseobacterium*	Chryseobacterium (100.0%)	Species****
bin.2	98.51	0.42	36844	3101576	Deinococcus	Deinococcus grandis*	Deinococcus (100.0%)	Subspecies****
bin.7	97.75	0	16123	2650064	Moraxellaceae	Psychrobacter sp. PRwf-1*	NA	Subspecies****
bin.3	97.41	1.33	32961	4014303	Neisseriales	Pseudogulbenkiania*	NA	Species****
bin.11	95.65	0.92	69243	2786419	Moraxellaceae	Psychrobacter*	NA	Species****
bin.10	95.12	0	13409	2459723	Deinococcaceae	Deinococcus*	NA	Species****
bin.12	93.14	0.85	24793	2851493	Deinococcaceae	Deinococcus*	NA	Species****
bin.6	88.74	1.45	7283	1988194	Micrococcales	Arthrobacter*	NA	Species****
bin.13	77.11	0.64	3045	2627969	Deinococcaceae	Deinococcus*	NA	Species****
bin.5	74.13	0.61	24837	1635952	Moraxellaceae	unclassified Moraxellaceae*	Alkanindiges (99%)	Species****
bin.8	67.76	0	10934	2837743	Deinococcaceae	Deinococcus*	NA	Species****
bin.9	61.13	0.30	2210	2110411	Janthinobacterium	Janthinobacterium sp. SNU WT3***	NA	Subspecies****

MAG name indicates the name given to that bin for this study (e.g., in Figure 1). The highest taxonomic rank with p-value ≤ 0.5 is shown in MiGA ID. RPD ID is the result of the identification analysis using rRNA genes (16S) implemented in MiGA; % indicates confidence in identification. Taxonomic novelty is a MiGA analysis that indicates the taxonomic rank at which the MAG represents a novel taxon with respect to the NCBI Genome database; highest taxonomic rank with p-value ≤ 0.01 are shown. Significance at p-value below: *0.5, ***0.05, ****0.01.

**FIGURE 2:**
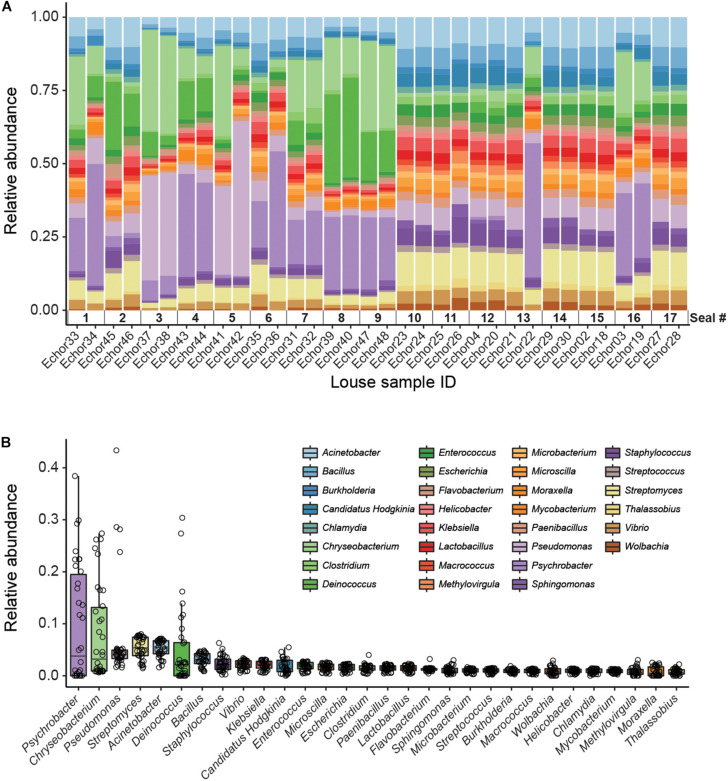
Kaiju data (genus level). **(A)** Stacked bar plot showing bacterial relative abundances in each seal louse sample. Note that samples are sorted according to host individual (i.e., samples from the same host are next to each other). **(B)** Boxplot summarizing the relative abundance of each taxon across all louse samples. Individual points (horizontally jittered) depict the relative abundance of each taxon in each sample.

Kaiju analyses recovered a higher diversity of microorganisms than did the genome-resolved approach (Figure 2 and Supplementary Figure 2). These differences are likely because of the quality-filtering parameters used in the genome-resolved metagenomics pipeline (i.e., these taxa may have been discarded because the completeness values of their bins were lower than 50% and/or their contamination values were higher than 10%). Nevertheless, bacterial taxa found in the genome-resolved metagenomic approach were generally found also in Kaiju and with similar relative abundances (Figure 2), and a similar pattern was found also at the species level (Supplementary Figure 2).

**FIGURE 3:**
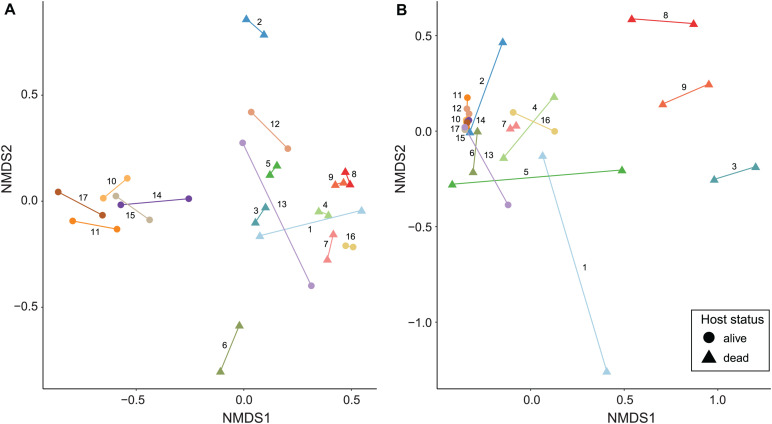
NMDS ordinations of seal louse microbiomes based on Bray–Curtis dissimilarity matrices. **(A)** MAG matrix (stress = 0.132), and **(B)** Kaiju matrix (species level, stress = 0.081). Lice originating from the same seal individual are colored similarly and connected by a line, numbers next to lines refer to seal individuals in Figures 1, 2.

Non-metric multidimensional scaling ordinations and PERMANOVA analyses show a major role of infrapopulation identity in explaining microbiome composition for both quantitative and presence–absence data. In the genome-resolved metagenomic dissimilarity matrices, the Bray–Curtis–based NMDS ordination evidenced a strong pattern of clustering by infrapopulation identity (Figure 3A). This pattern was not noticeable in the Jaccard-based NMDS ordination because dissimilarity was too low among samples (Supplementary Figure 3A). PERMANOVA analyses indicated that most (>84% in all cases) of the variance was explained by infrapopulation identity (PERMANOVA: Bray–Curtis, *R*^2^ = 0.857, *F* = 6.419, *P* = 0.001, Figure 3A; Jaccard, *R*^2^ = 0.842, *F* = 5.671, *P* = 0.001; Supplementary Figure 3A). The analyses of differences in pairwise distances showed highly consistent results, as pairwise distances between samples from the same infrapopulations were lower than the median of among-infrapopulation pairwise comparisons in 16 out of 17 infrapopulations (94%; Sign test: *P* < 0.001; Supplementary Figures 4, 5). Results from the simulations were in line with the results of the regular model, and thus support that our results were not biased by the sampling design [PERMANOVA: Bray–Curtis, R^2^ (min = 0.65, max = 0.98, mean = 0.78); P (min = 0.001, max = 0.019, *n* < 0.05 = 10/10); Jaccard, R^2^ (min = 0.66, max = 1, mean = 0.86), P (min = 0.001, max = 0.106, *n* < 0.05 = 5/7)]. From all the additional factors examined, only host status (i.e., dead, alive) explained a significant amount of variance (Table 2). Including host status in PERMANOVA analyses did not alter the results on the major influence of host identity in explaining microbiome composition (PERMANOVA: Host identity, Bray–Curtis, *R*^2^ = 0.57, *F* = 4.58, *P* = 0.001; Jaccard, *R*^2^ = 0.71, *F* = 5.09, *P* = 0.002).

**TABLE 2:** PERMANOVA results from the factors in addition to infrapopulation that were evaluated to potentially influence microbiome variation among samples.

*Data type*	*MAGs*						*Kaiju*					
*Dissimilarity distance*	Bray–Curtis			Jaccard			Bray–Curtis			Jaccard		
*Statistic Factor*	R^2/^ρ	F	P	R^2/^ρ	F	P	R^2/^ρ	F	P	R^2/^ρ	F	P
*Host status*	0.28	12.72	0.001	0.13	4.93	0.002	0.22	9.03	0.001	0.21	8.73	0.001
*Louse sex*	0.08	0.9	0.554	0.03	0.28	0.867	0.08	0.81	0.564	0.08	0.88	0.497
*Sequencing lane*	0.01	0.38	0.878	0	0.01	1	0.01	0.35	0.859	0.01	0.4	0.825
*Locality*	−0.09; −0.09; −0.09	–	0.875; 0.887; 0	−0.29; −0.29; −0.29	–	0.97; 0.978; 0	0.04; 0.04; 0.04	–	0.564; 0.579; 0	−0.03; −0.03; −0.03	–	0.534; 0.549; 0

*Note that the influence of locality was tested using multiple Mantel tests, and therefore ρ (min, max, and mean) and P (min, max, n < 0.05) simulation values are given (see section “Materials and Methods” for further details).*

Similarly, in Kaiju matrices collapsed at the species level, NMDS ordinations showed a pattern of clustering by infrapopulation identity (Figure 3B and Supplementary Figure 3B). In the same vein, most (>80% in all cases) of the variance was also explained by infrapopulation identity (PERMANOVA: Bray–Curtis, *R*^2^ = 0.804, *F* = 4.346, *P* = 0.001, Figure 3B; Jaccard, *R*^2^ = 0.803, *F* = 4.319, *P* = 0.001; Supplementary Figure 3B). The analyses of differences in pairwise distances again supported the results: pairwise distances between samples from the same infrapopulations were lower than the median of among infrapopulation pairwise comparisons in 15 out of 17 infrapopulations (88%; Sign test: *P* < 0.01; Supplementary Figures 5, 6). Furthermore, results from simulations were similar [PERMANOVA: Bray–Curtis, R^2^ (min = 0.62, max = 0.88, mean = 0.75); P (min = 0.003, max = 0.058, *n* < 0.05 = 9/10); Jaccard, R^2^ (min = 0.63, max = 0.95, mean = 0.76), P (min = 0.002, max = 0.09, *n* < 0.05 = 9/10)]. Of all the other factors examined, only host status explained a significant amount of variance (Table 2). PERMANOVA analysis accounting for host status did not alter the relevance of host identity in explaining a significant amount of variance (PERMANOVA: Bray–Curtis, *R*^2^ = 0.52, *F* = 1.78, *P* = 0.007; Jaccard, *R*^2^ = 0.59, *F* = 3.37, *P* = 0.001).

Furthermore, results were consistent when using matrices collapsed at the genus level. Samples appeared clustered by infrapopulation identity in NMDS ordinations (Supplementary Figures 7A,B) and > 77% of variance was explained in all cases by this factor (PERMANOVA: Bray–Curtis, *R*^2^ = 0.865, *F* = 6.804, *P* = 0.001, Supplementary Figure 7A; Jaccard, *R*^2^ = 0.774, *F* = 3.634, *P* = 0.001; Supplementary Figure 7B). Once again, results from simulations were similar [PERMANOVA: Bray–Curtis, R^2^ (min = 0.68, max = 0.96, mean = 0.8); P (min = 0.002, max = 0.073, *n* < 0.05 = 9/10); Jaccard, R^2^ (min = 0.54, max = 0.86, mean = 0.73), P (min = 0.003, max = 0.061, *n* < 0.05 = 9/10)]. Additionally, of all the others factors examined, only host status explained a significant amount of variance [PERMANOVA: Bray–Curtis, Host status: *R*^2^ = 0.3, *F* = 14, *P* = 0.001, Louse sex: *R*^2^ = 0.05, *F* = 0.51, *P* = 0.851, Sequencing lane: *R*^2^ = 0.01, *F* = 0.39, *P* = 0.753; Jaccard, Host-status: *R*^2^ = 0.18, *F* = 7.19, *P* = 0.002, Louse sex: *R*^2^ = 0.07, *F* = 0.75, *P* = 0.53, Sequencing lane: *R*^2^ = 0.01, *F* = 0.40, *P* = 0.75; Mantel test, locality, Bray–Curtis: ρ (min = 0.09, max = 0.09, mean = 0.09), P (min = 0.720, max = 0.734, *n* < 0.05 = 0/10); Jaccard: ρ (min = 0.02, max = 0.02, mean = 0.02), P (min = 0.404, max = 0.425, *n* < 0.05 = 0/10)]. Likewise, PERMANOVA analysis accounting for host status did not alter the results on the relevance of host identity (PERMANOVA: Bray–Curtis, *R*^2^ = 0.56, *F* = 4.73, *P* = 0.001; Jaccard, *R*^2^ = 0.59, *F* = 2.96, *P* = 0.001).

## Discussion

Two different metagenomic approaches support a major role of infrapopulation identity (ringed seal host individual) in explaining microbiome variation among individuals of the seal louse. In addition, highly similar results were found for approaches using either presence–absence or quantitative matrices, suggesting that not only is bacterial composition, but also bacterial abundance explained by infrapopulation identity. Our analyses were done on whole louse individuals and, thus, we cannot confidently differentiate between bacterial taxa inhabiting the lice (e.g., *Wolbachia* or *Hodgkinia*) from transient taxa present in the host blood meal (e.g., *Chlamydia*). Nevertheless, in line with current evidence on the determinants of microbiome composition of bloodsucking parasites, the louse blood meal from individual seals is the most likely candidate in explaining the patterns of microbiome variation across the focal louse infrapopulations. Indeed, many of the taxa found in our analyses have already been found in other bloodsucking parasites, thus supporting the influence of blood in shaping the composition of parasite microbiomes studied here (Jiménez-Cortés et al., 2018).

However, other factors in addition to blood may have contributed to the similarity of microbiomes between individual lice from the same seal host individual. Some similarity may have arisen from shared environmental bacteria, i.e., those on the surface of the louse from a shared environment (skin and fur of the host), or contamination between louse individuals in screw-cap tubes, and not filtered by our decontamination procedures. While some potential contamination sources are nearly impossible to avoid, possible contamination between louse individuals in screw-cap tubes could have been avoided in this study should the louse from the same individuals have been placed in separate screw-cap tubes. We believe it seems unlikely, especially for some bacterial taxa (e.g., gut bacteria adhered to the gut epithelial cells; Narasimhan and Fikrig, 2015), that once in ethanol, these bacteria could have gone out of the louse individuals and reached the other louse interior. Nevertheless, our ethanol rinses, procedures to extract DNA (i.e., crashing whole louse individuals), and the bioinformatic decontamination filtering ensure this process does not mainly drive our results. There may also be insect-specific bacterial taxa, independent from the host blood, that are shared horizontally between individual lice from the same infrapopulation. Finally, louse infrapopulations are known to typically be highly inbred, with a high level of relatedness between individuals (Koop et al., 2014; DiBlasi et al., 2018; Virrueta Herrera et al., in prep.). It may be that genetic factors of the lice interact with the microbiome to produce a specific composition (Blekhman et al., 2015; Dobson et al., 2015; Suzuki et al., 2019).

Our results are congruent with previous findings on the influence of host blood on microbiomes of bloodsucking parasites. Specifically, several studies have found a major role of the specific host species from which a blood meal is taken in shaping microbiomes of other bloodsucking organisms, such as ticks (*Ixodes scapularis, Ixodes pacificus*) and mosquitoes (*Aedes aegypti*) (Swei and Kwan, 2017; Landesman et al., 2019; Muturi et al., 2019). Furthermore, Landesman et al. (2019) showed that microbiomes of deer tick (*Ixodes scapularis*) nymphs were largely explained by the individual hosts of the tick, a result similar to the one obtained here. Interestingly, in that study, the percentage of variation explained was considerably lower (45%) than that found here (>77%). It may be that differences in parasite ecology, such as the whether the parasite is a permanent or a recurrent feeder (as are both the case in sucking lice) may modulate the extent to which host blood shapes parasite microbiomes. The differences in the proportion of variance explained by infrapopulation identity between the two studies could also be due to differences in experimental design, such as the number of sampled infrapopulations (3 in ticks, and 17 in the seal lice here) and whether the sample design is balanced (i.e., the same number of individual parasites sampled per infrapopulation).

The knowledge that intraspecific variation in the blood of seals may lead to similarity of the microbiomes of lice feeding on the same host individual can potentially provide new insights into the influence of host blood on parasites. At least two not necessarily mutually exclusive processes may explain the influence of a host individual’s blood on louse microbiomes. First, the blood from a particular host individual may contain a specific composition of bacterial loads that enter the louse during feeding. Indeed, anopluran lice might have a high likelihood of being colonized by bacteria from host blood because they do not possess a peritrophic membrane, an extracellular layer in the midgut that is composed of chitin, proteoglycans, and proteins, which in most other insects surrounds the ingested food bolus and separates the gut content, including bacteria, from the epithelium (Terra, 2001; Waniek, 2009). Indeed, the idea that a lack of a peritrophic membrane facilitates colonization of blood-feeding parasites by bacteria present in the host blood has potentially also been supported by work on mouse fleas (*Rhadinopsylla dahurica*), which also lack this membrane (Li et al., 2018). In this case, there was evidence of homogenization (i.e., similar bacterial communities) between the host blood and the parasite (whole flea individuals). The lack of a peritrophic membrane is often associated with permanent parasites, such as blood-feeding lice, for which the continual availability of food means that there is less selection for efficient digestion. Therefore, the presence versus absence of a peritrophic membrane may explain the differences between lice and ticks (of which the latter possess a peritrophic membrane) with regards to the influence of host blood on the composition of the parasite microbiome.

A second possibility that could explain why host blood may influence louse microbiome composition is that the conditions during blood digestion may alter bacterial taxa that were already present in the louse. The specifics of blood digestion may have an individual host-specific signature. Specifically, catabolism of blood meal leads to the generation of reactive oxygen species that are known to alter the midgut bacterial composition and diversity of bloodsucking parasites (Souza et al., 1997; Wang et al., 2011; Muturi et al., 2019). Also, the blood of different host species is known to differ in composition (e.g., total protein, hemoglobin, and hematocrit content), and these differences may lead to a differential proliferation of microbial taxa during digestion by parasites (Souza et al., 1997; Wang et al., 2011; Muturi et al., 2019). It may be the case that differences in blood composition among individuals even within the same host species may shape the bacterial composition of lice in a manner that is specific to host individuals.

**FIGURE 4:**
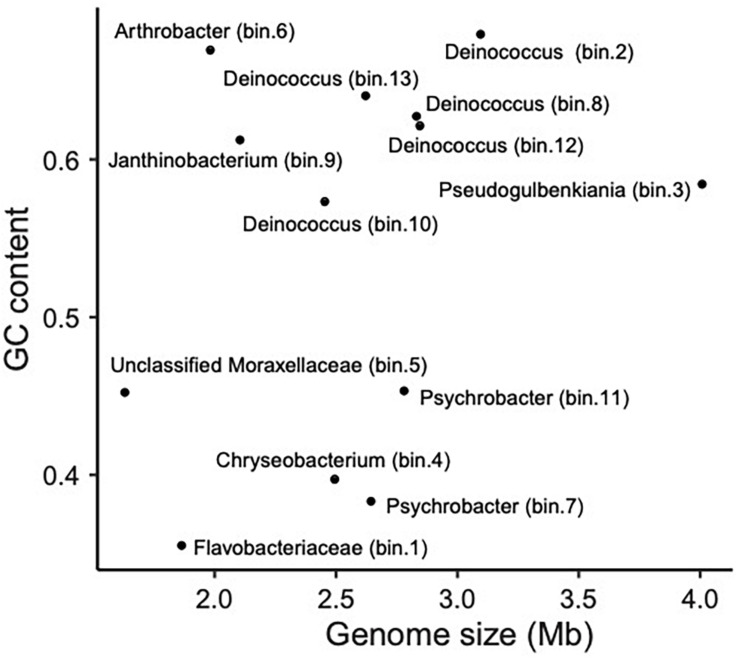
Scatter plot showing the relationship between genome size (Mb) and GC content (i.e., proportion of G and C sites) for sequenced MAGs.

Bloodsucking organisms, and anopluran lice in particular, are well known to rely on mutualistic endosymbionts to complement deficiencies in their diet (Perotti et al., 2008; Boyd and Reed, 2012; Boyd et al., 2017; Jiménez-Cortés et al., 2018). Notwithstanding that several of the bacterial taxa we found may not be stable inhabitants of lice, we found evidence for the presence of several louse-specific bacterial taxa (i.e., taxa that are highly unlikely to come from environmental contamination). These include the obligate intracellular arthropod bacteria *Wolbachia* (Werren, 1997) and *Hodgkinia* (for which only endosymbionts of cicadas are known; McCutcheon et al., 2009). Accordingly, we explored our MAGs for genome characteristics typical of endosymbionts. In particular, because endosymbiont genomes typically are small and have an AT bias, we explored the relative position of the observed MAGs in a “Genome size ∼ GC content” correlation plot (Wernegreen, 2015; Figure 4). Bin 1 appears to be the best candidate to be a mutualistic endosymbiont, according to its relative position in the correlation plot. This MAG was 100% complete (according to CheckM; Parks et al., 2015), detected in most samples (prevalence = 71%; similar endosymbiont prevalences have been found in Allen et al., 2016 and Říhová et al., 2019), and classified with confidence as Flavobacteriaceae. MiGA analyses suggest it may even belong to *Chryseobacterium* (*p*-value 0.585). Endosymbionts belonging to *Chryseobacterium* are known in other arthropods (e.g., termites, mosquitoes, cockroaches, and ticks; Eutick et al., 1978; Dugas et al., 2001; Campbell et al., 2004; Montasser, 2005; Burešová et al., 2006). Additionally, we conducted a preliminary investigation of the metabolic capabilities of this bacterium by investigating the completeness of metabolic pathways using GhostKOALA (Kanehisa et al., 2016) and KEGG-Decoder (Graham et al., 2018). This MAG has complete routes for synthesis of vitamin B (riboflavin), an essential amino acid (lysine), and several non-essential amino acids (e.g., serine; see Supplementary Table 4), as well as many fully or partially missing routes that may be redundant or potentially shared (or synthesized along) with the louse (Supplementary Table 4).

Overall, these results are congruent with what has been found for endosymbionts of bloodsucking parasites (Moriyama et al., 2015; Boyd et al., 2016; Santos-Garcia et al., 2017; Duron et al., 2018). Nevertheless, it is worth mentioning that the relative abundance of this putative endosymbiont MAG was the lowest among all the MAGs studied here. However, in contrast to what it is known for other bloodsucking parasites (e.g., ticks; Narasimhan and Fikrig, 2015), little is known in lice about the abundance and prevalence patterns of the microbiome of different organs and systems. In addition, apart from this study, whole-genome metagenomic data from different individuals are not available. Further research on the individual-level microbiomes of lice is needed to understand the relative abundances of bacteria in lice.

Another anopluran pinniped louse (*Proechinophthirus fluctus*) has been found to have a *Sodalis* endosymbiont (Boyd et al., 2016), but we found no evidence of *Sodalis* in *Echinophthirius horridus*. Other species of Anoplura have yet other endosymbionts (Boyd et al., 2017; Říhová et al., 2017, 2019), suggesting that endosymbiont replacement is an ongoing and relatively common process within the order. Population-scale studies are needed to better understand endosymbiont dynamics in populations of lice. Also, phylogenomic studies aiming at elucidating the phylogenetic placement of the potentially mutualistic *Chryseobacterium* found here and studies using fluorescence *in situ* hybridization (FISH) to ascertain its location in louse individuals are needed to get deeper insight into the interaction of this bacterium with *E. horridus*.

Lastly, the methodology used in this study (i.e., a dual metagenomic approach that combines genome-resolved metagenomics with metagenomic classification tools and state-of-the-art bioinformatic decontamination procedures) opens the door to further studies of the microbiomes of both parasites and free-living organisms for which WGS data are available. Here, this approach allowed us to characterize the variation of microbiomes among individuals of the same parasite species, and to identify factors underlying the observed variation. We were also able to identify potential endosymbionts and to recover high-quality genomic data from them. Interestingly, in the current field of genomics, excessive accumulation of data and the resultant ever-increasing demand for data-storage capacity are worrying trends (Stephens et al., 2015). Thus, the possibility of using the same genome-level sequence datasets to address multiple different research questions (e.g., data generated for population-genomic analyses and cophylogenomics later leveraged to investigate introgression dynamics; Doña et al., 2020) and in different contexts (e.g., host-derived population-genomic data to infer bacterial composition; Hooper et al., 2019, this study) allows for more efficient use of existing genomic resources.

## Data Availability Statement

Raw sequence reads for all samples are available at SRA (see Supplementary Table 1). Metagenomic assemblies and FastQC reports (before and after preprocessing) are available at Figshare (doi: 10.6084/m9.figshare.12366575). Metagenomic assemblies are also available from NCBI Genome (SAMN18543074–SAMN18543086).

## Ethics Statement

Telemetry studies have been approved by the local environmental authority Centre for Economic Development, Transport and the Environment (permit numbers: ESAELY/433/07.01/2012 and ESA-2008-L-519-254) and the Animal Experiment Board in Finland (permit numbers: ESAVI/8269/04.10.07/2013 and ESAVI-2010-08380/Ym-23).

## Author Contributions

JD, SV, and KJ conceived the study. TN and MK obtained samples. SV and KJ collected the data. JD analyzed the data and wrote the manuscript. TN, MK, and KJ obtained financial support for the project. All authors contributed to editing the manuscript.

## Conflict of Interest

The authors declare that the research was conducted in the absence of any commercial or financial relationships that could be construed as a potential conflict of interest.

## Footnotes

1 https://sourceforge.net/projects/bbmap/
