# Supplementary figures and images for: Patterns of Microbiome Variation Among Infrapopulations of Permanent Bloodsucking Parasites
Source: Front Microbiol. 2021 Apr 16;12:642543. doi: 10.3389/fmicb.2021.642543 (PMC8085356; doi:10.3389/fmicb.2021.642543)

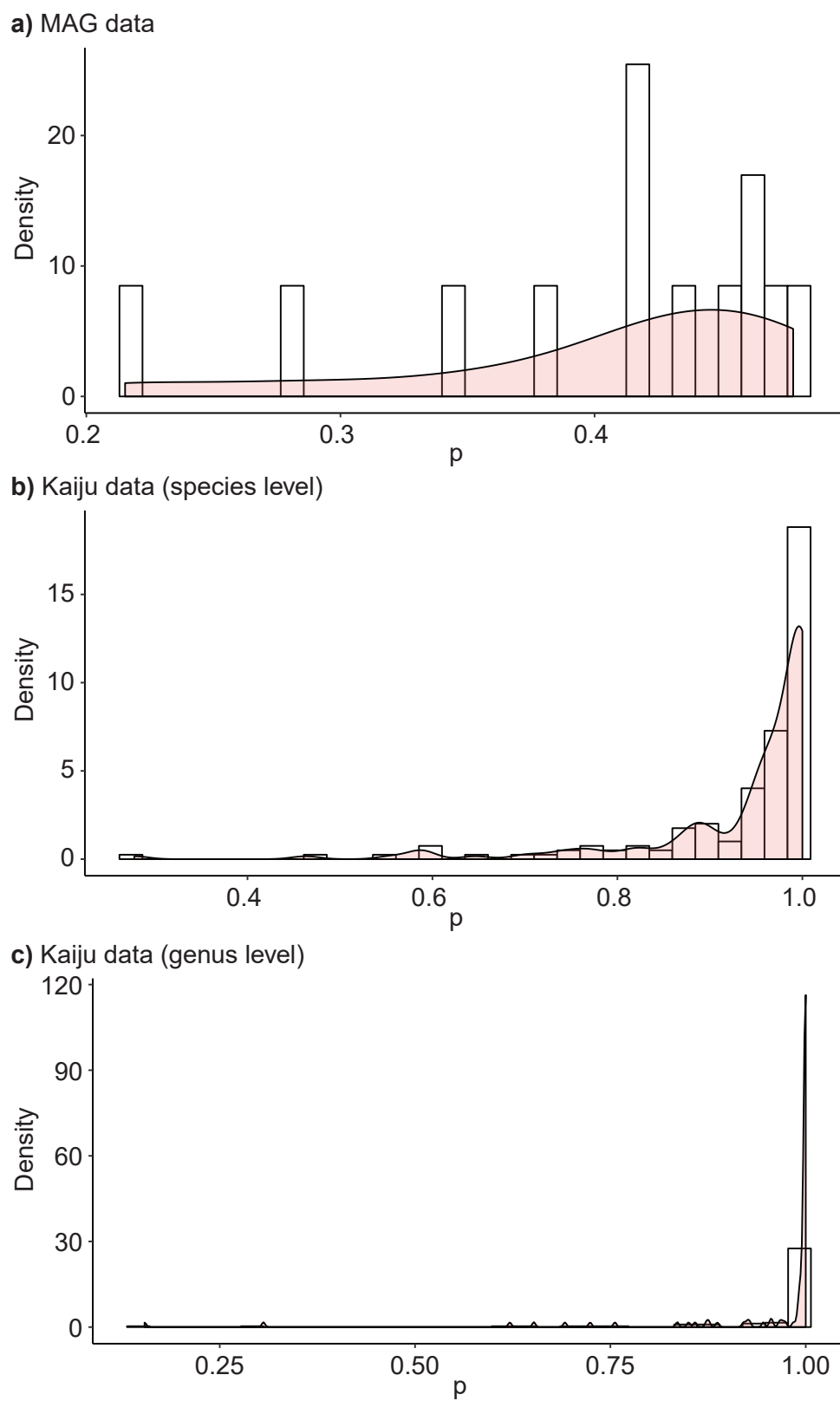

**Figure S1.** Score distributions for each dataset assigned by Decontam.

Supplement: Supplementary file 1 [file Data_Sheet_1.PDF]
