# Supplementary material for: Patterns of Microbiome Variation Among Infrapopulations of Permanent Bloodsucking Parasites
Source: Front Microbiol. 2021 Apr 16;12:642543. doi: 10.3389/fmicb.2021.642543 (PMC8085356; doi:10.3389/fmicb.2021.642543)
Supplement: Supplementary file 2 [file Data_Sheet_2.pdf]

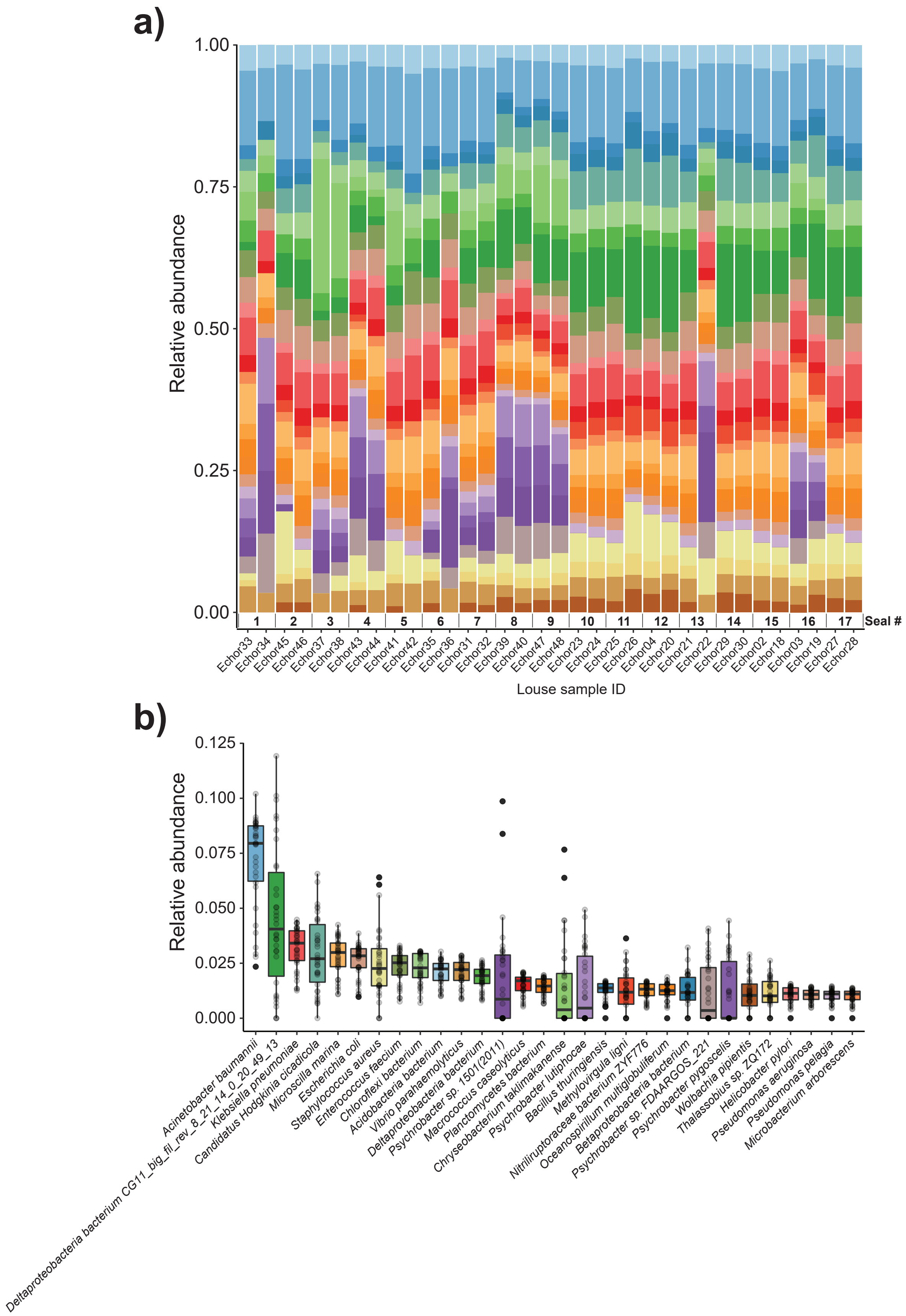

Figure S2. Kaiju data (species level). (a) Stacked bar plot showing bacterial relative abundances in each seal louse sample. Note that samples are sorted according to host individual (i.e., samples from the same seal are next to each other). (b) Boxplots summarizing the relative abundance of each taxon across all louse samples.
