# Supplementary material for: Patterns of Microbiome Variation Among Infrapopulations of Permanent Bloodsucking Parasites
Source: Front Microbiol. 2021 Apr 16;12:642543. doi: 10.3389/fmicb.2021.642543 (PMC8085356; doi:10.3389/fmicb.2021.642543)
Supplement: Supplementary file 3 [file Data_Sheet_3.pdf]

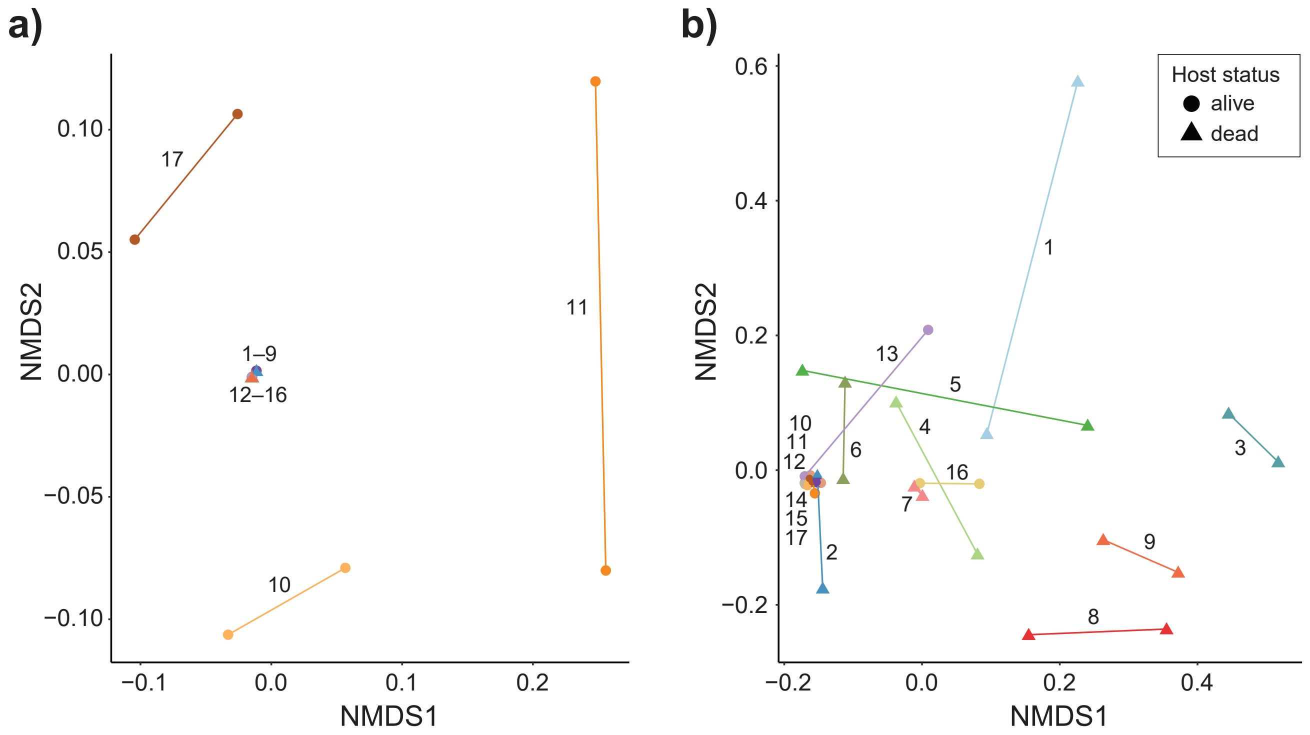

Figure S3. NMDS ordinations of seal louse microbiomes based on Jaccard dissimilarity matrices calculated on the basis of (a) MAG data, and (b) Kaiju data (species level). Lice originating from the same seal host individual are colored similarly and connected by a line with the seal number.
