# Supplementary material for: Patterns of Microbiome Variation Among Infrapopulations of Permanent Bloodsucking Parasites
Source: Front Microbiol. 2021 Apr 16;12:642543. doi: 10.3389/fmicb.2021.642543 (PMC8085356; doi:10.3389/fmicb.2021.642543)
Supplement: Supplementary file 4 [file Data_Sheet_4.pdf]

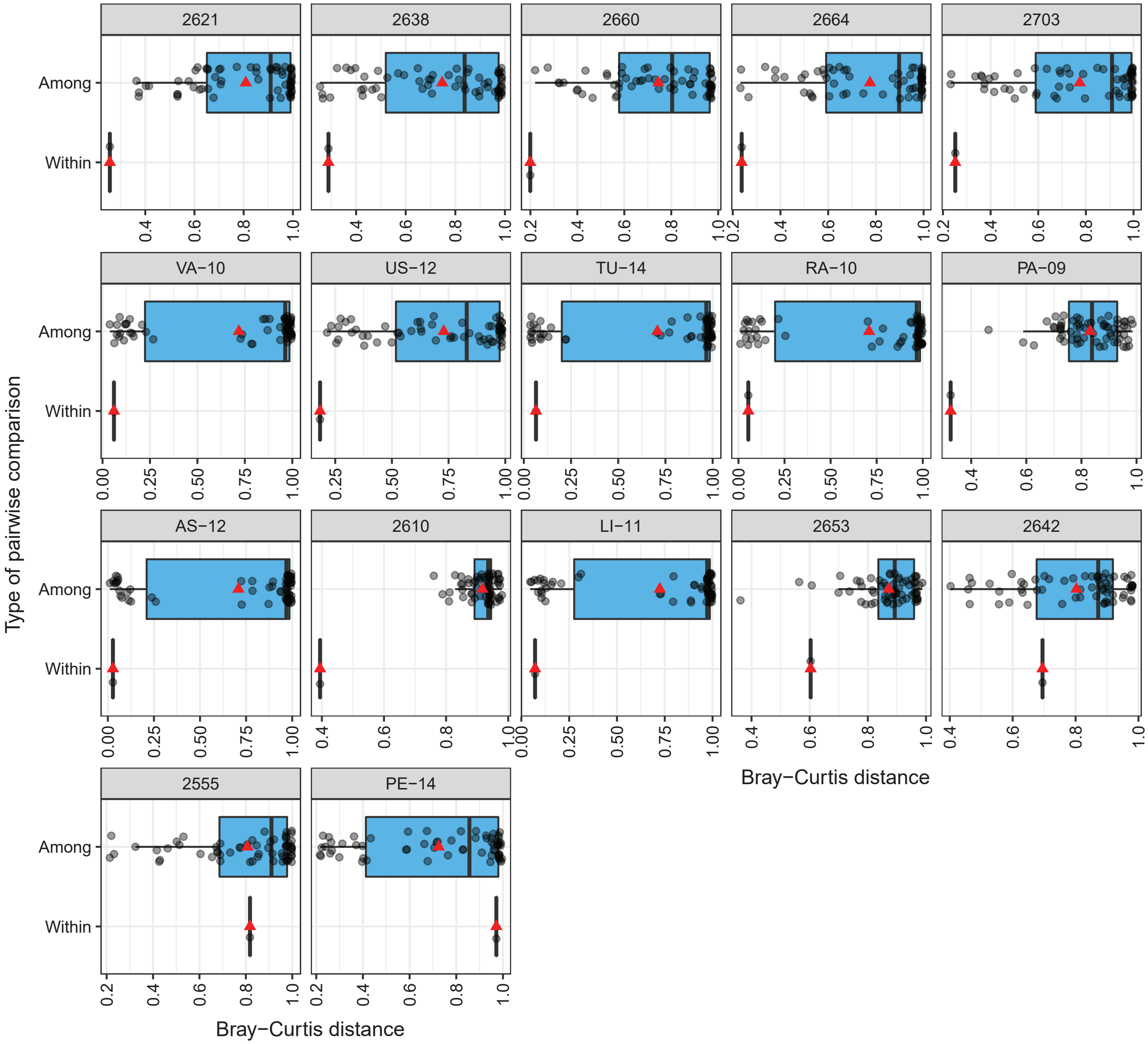

Figure S4. Pairwise distances among samples (MAG data). Horizontal solid lines show medians, red triangles show means.
