# Supplementary material for: Patterns of Microbiome Variation Among Infrapopulations of Permanent Bloodsucking Parasites
Source: Front Microbiol. 2021 Apr 16;12:642543. doi: 10.3389/fmicb.2021.642543 (PMC8085356; doi:10.3389/fmicb.2021.642543)
Supplement: Supplementary file 5 [file Data_Sheet_5.pdf]

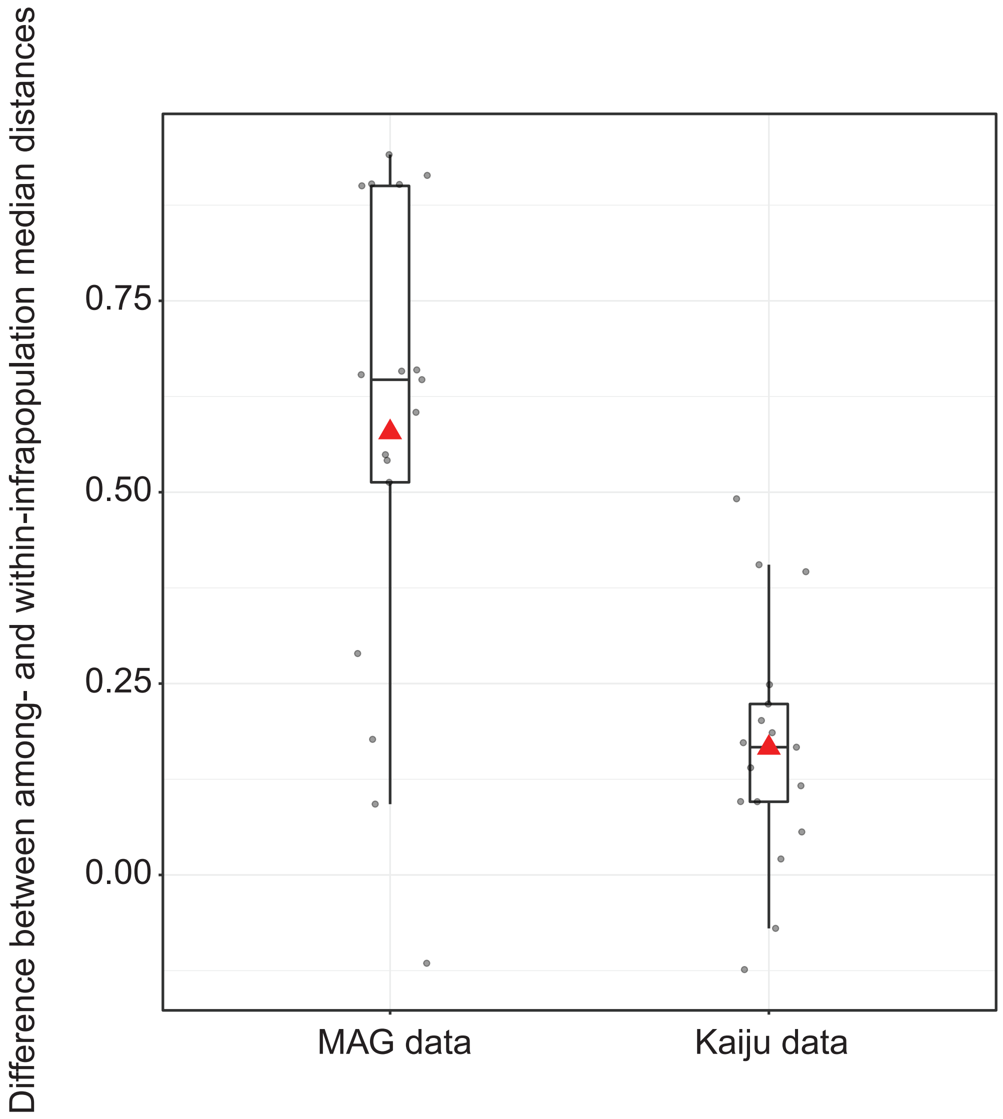

Figure S5. Boxplots showing the difference between among- and within-infrapopulation median distances (Bray–Curtis dissimilarity) for the MAG (left) and Kaiju (right) data. Horizontal solid lines show medians and red triangles means.
