# Supplementary material for: Patterns of Microbiome Variation Among Infrapopulations of Permanent Bloodsucking Parasites
Source: Front Microbiol. 2021 Apr 16;12:642543. doi: 10.3389/fmicb.2021.642543 (PMC8085356; doi:10.3389/fmicb.2021.642543)
Supplement: Supplementary file 7 [file Data_Sheet_7.pdf]

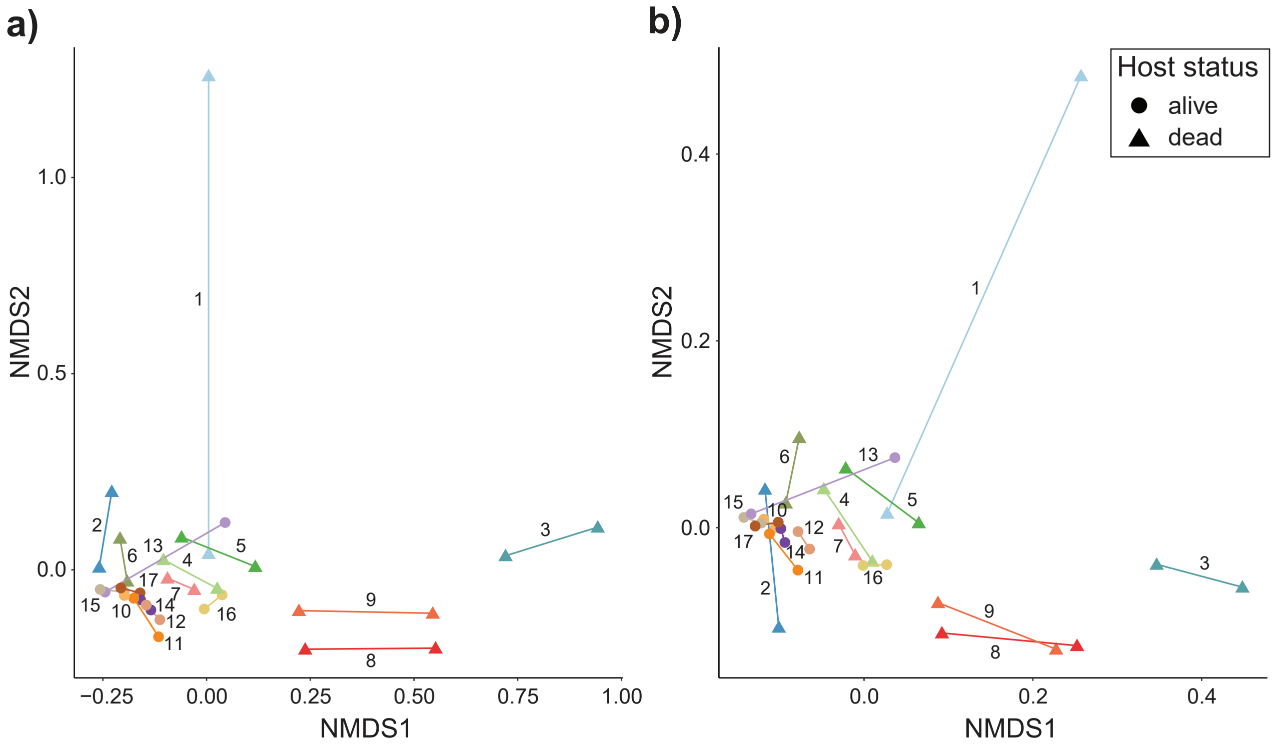

Figure S7. NMDS ordinations of seal louse microbiomes based on (a) Bray–Curtis dissimilarity and (b) Jaccard distances calculated from Kaiju data collapsed to the genus level. Lice originating from the same seal host individual are colored similarly and connected by a line with the seal number.
